# Long-term BMI and growth profiles in offspring of women with gestational diabetes

**DOI:** 10.1007/s00125-018-4584-4

**Published:** 2018-02-28

**Authors:** Nurah M. Hammoud, Gerard H. A. Visser, Lenie van Rossem, Douwe H. Biesma, Jan M. Wit, Harold W. de Valk

**Affiliations:** 1 Department of Obstetrics, Division Woman & Baby, the University Medical Center Utrecht, Heidelberglaan 100, Huispostnummer KE.04.123.1, P.O. Box 85090, 3508 AB Utrecht, the Netherlands; 2 Department of Internal Medicine and Infectious Diseases, the University Medical Center Utrecht, Utrecht, the Netherlands; 3 Julius Center for Health Sciences and Primary Care, University Medical Center Utrecht, Utrecht, the Netherlands; 4 Department of Pediatrics, Leiden University Medical Center, Leiden, the Netherlands

**Keywords:** Body mass index, Childhood obesity, Diabetic pregnancy, Gestational diabetes mellitus, Growth trajectories, Longitudinal follow-up, Obstetrics, Offspring

## Abstract

**Aims/hypothesis:**

Gestational diabetes mellitus (GDM) is reported to be associated with childhood obesity, however the magnitude of this association and relation to intrauterine growth is uncertain. We, therefore, aimed to assess whether the growth trajectories of large for gestational age (LGA) and non-LGA offspring of mothers with GDM (OGDM) are different until early adolescence. We also aimed to explore whether growth trajectories of OGDM differ from those of offspring of mothers with type 1 or 2 diabetes (ODM1, ODM2).

**Methods:**

We studied height and BMI standard deviation score (SDS) of the OGDM group, up to the age of 14 years, with subgroup analysis comparing LGA with non-LGA at birth as a reflection of the intrauterine environment. All mothers with GDM who delivered at the University Medical Center Utrecht between 1990 and 2006 were contacted to participate; informed consent was received for 104 OGDM of 93 mothers. Offspring data were collected through Dutch infant welfare centres. Recorded height and weight were converted to BMI and age- and sex-specific SDS values for Dutch children. Additionally, we compared the OGDM group with ODM1 and ODM2 groups in order to identify those offspring with the highest risk of becoming overweight. Growth trajectories were compared between non-LGA and LGA OGDM and between OGDM, ODM1 and ODM2, using a random-effects model. In the longitudinal follow-up a mean of 7.4 ± 2 measurements per infant were available.

**Results:**

Mothers had a prepregnancy BMI of 25.8 kg/m^2^ and 24% of their infants were LGA at birth. Heights of OGDM were no different from those of the Dutch Growth Study. Non-LGA OGDM showed a BMI SDS comparable with that of the reference population, with a slight increase in early adolescence. LGA OGDM had a higher BMI SDS trajectory than non-LGA OGDM and the reference population, which plateaued at around 10 years of age. Comparison of growth trajectories of OGDM, ODM1 and ODM2 showed ODM2 to have the highest trajectory followed by ODM1 and OGDM, with the LGA counterparts of all three offspring groups in the highest BMI SDS ranges.

**Conclusions/interpretation:**

Until early adolescence, OGDM have a BMI that is 0.5 SDS higher than that of the Dutch background population. LGA OGDM appear to be at particularly higher risk of being overweight in adolescence compared with non-LGA OGDM, putting them also at a higher lifetime risk of being overweight and developing obesity. ODM2 showed the highest BMI SDS values and had an average BMI SDS of +1.6 until the age of 14, when it became +2 SD. These results emphasize the importance of adequate recognition and timely treatment of maternal gestational diabetes to prevent fetal macrosomia in obstetrics.


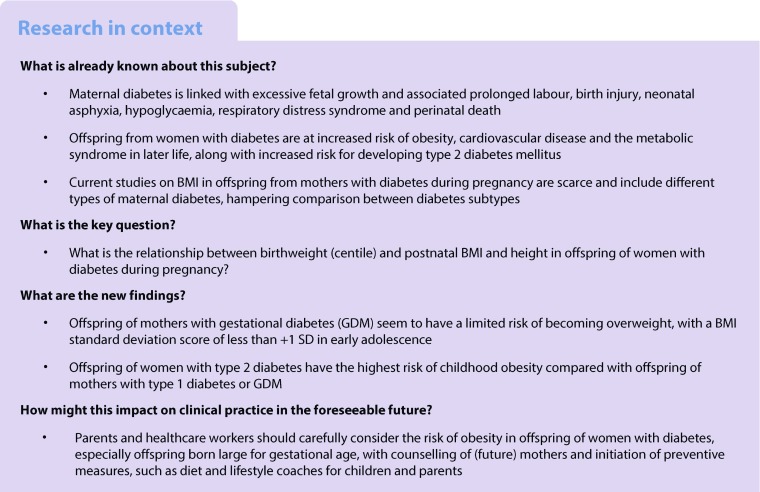


## Introduction

Gestational diabetes (GDM) is defined as any degree of glucose intolerance that is diagnosed in the second or third trimester of pregnancy and that is not clearly overt diabetes [1]. The prevalence of GDM is rising worldwide [2, 3] and the condition is associated with adverse pregnancy outcomes such as an increased risk of large-for-gestational-age (LGA) neonates and birth complications [4]. These LGA infants have an increased fat mass and decreased lean body mass compared with LGA infants from non-diabetic women [5]; their intrauterine growth is disproportionate, with an increased abdominal circumference compared with the head circumference ratio [6]. In adolescence, offspring of mothers with GDM (OGDM) have been shown to have an increased risk of abnormal glucose metabolism and hypertension [7, 8]. It is not known whether intrauterine adiposity adds to postnatal adiposity and subsequent health issues.

To anticipate childhood obesity and formulate preventive strategies, it is important to perform longitudinal studies of BMI. Studies of BMI in OGDM have mostly been cross-sectional in design [7–16] and only a few longitudinal studies have been performed in GDM [17–20]. The cross-sectional cohorts showed an increased risk for developing adiposity compared with children not exposed in utero to diabetes [7, 10, 12]; even mild GDM, untreated or treated only with dietary intervention, was associated with an increase in offspring obesity between the ages of 5 and 10 years [11]. Pooling the different cross-sectional studies is difficult due to different definitions of endpoints used (obesity, BMI or BMI *z* score) [7, 8, 10, 13]. Also, the definitions used for obesity differed [10, 13]. The available longitudinal data are unsuitable for comparison due to differences in methodology: different age categories and follow-up periods (age 6 months to 13 years) and different statistical methods and different assessments of body mass (BMI, BMI standard deviation score [SDS], quintiles and skinfold thickness) [17–21]. Additionally, different types of maternal diabetes were included in most studies [14, 15, 21, 22]. The available evidence on longitudinal BMI data in OGDM show a higher childhood BMI up to the age of 13 years compared with offspring of non-diabetic pregnancies [21], and a higher childhood BMI SDS up to age 8 and 14 years compared with background reference populations [18, 20]. This indicates that OGDM have an increased risk for higher BMI or frank obesity when reaching late childhood / early adolescence.

Little is known how being born LGA influences postnatal growth although this is a question commonly posed by parents. Only a single longitudinal study investigated LGA neonates as a subgroup and showed that these infants do indeed have the highest BMI at age 4–7 years [16]. Unfortunately, there are no data available on height development of OGDM. Height trajectories give an indication of the extent to which this variable influences overall BMI.

The current study was performed to answer two questions. First, to assess whether the growth trajectories of LGA and non-LGA OGDM are different until early adolescence, with LGA as a reflection of intrauterine adiposity. Second, to explore whether growth trajectories of OGDM differ from those of offspring of mothers with type 1 diabetes (ODM1) or offspring of mothers with type 2 diabetes (ODM2). Our data were compared with several nationwide Dutch growth studies (mainly the Fifth Dutch Growth Study), which sampled and assessed children up to adolescence. Since 1955, these studies have been performed in the Netherlands every 10–15 years and provide data on the prevalence rates of overweight and obesity.

## Methods

### Participants

The study group consisted of offspring of women with GDM who delivered in the University Medical Center, Utrecht, the Netherlands between 1990 and 2006. All women who delivered in this period were contacted in 2013. After consent was obtained, individual offspring growth charts from the Dutch infant welfare centres were retrieved. The parents were invited by mail to complete a questionnaire, including questions regarding maternal and paternal height, weight, comorbidities and ethnicity. Parents were asked to provide the most recent height and weight of the child, measured either by healthcare workers or themselves.

Infant weight and length were collected through Dutch infant welfare centres, which have a high coverage and record infant weight, supine length (until 2.0 years) and height (from 2.0 years onward) through standard protocols on specified dates between birth and age 4 years (1, 4, 6, 9, 11, 14, 18, 24, 36, 45 months). Thereafter, children are measured, by trained healthcare professionals in the school health service, at 5.5, 11 and 13 years of age, with a range of 1–2 years around these ages. Infants’ length and standing height were measured to the nearest 0.1 cm. Up to age 15 months, children were weighed naked. Older children were weighed wearing underwear only, on calibrated mechanical or electronic step scales. Weight was measured to the nearest 0.1 kg. The medical ethics committee of the University Medical Center Utrecht, Utrecht in the Netherlands (application no. 13/179; reference no. WAG/om/13/053639) approved this study (9 April 2013).

GDM was diagnosed in 79% of the women using the 75 g oral glucose tolerance test, and in the remaining participants through elevated fasting glucose levels or an abnormal glucose profile showing hyperglycaemia.

### Data collection

Baseline maternal characteristics at pregnancy and information on pregnancy outcomes were retrieved from records of the University Medical Center Utrecht. Parents provided information regarding their own current height and weight, educational status and current height and weight for each child when they completed the written questionnaire. Birthweight SDS was calculated as follows: (birthweight − mean birthweight for sex, parity and gestational age) / SD for sex, parity and gestational age, based on Dutch reference data [23]. LGA was defined as birthweight ≥90th percentile corrected for gestational age, sex and parity [23]. The conditional target height of offspring was calculated based on parental height according to Hermanussen and Cole [24] and adapted to Dutch growth standards [25]. Length and height of OGDM were expressed as SDS for age and sex based on the Fifth Dutch Growth Study performed in 2009 [26]. BMI was calculated from height and weight with the following formula: weight (kg) / (height [m])^2^. BMI was expressed as SDS for the 1980 nationwide growth study, in which SDS 0 equals the age- and sex-specific mean of the 1980 Dutch reference population [27]. These 1980 data are used as the Dutch normative standard for BMI, thus reflecting a degree of overweight and obesity in the study group. Our data were also compared with the 2009 Dutch BMI data to enable comparison with the current child population [28]. The values from the 2009 nationwide study were plotted in the BMI SDS graphs for visual comparison of our offspring from the diabetic pregnancies, to show the effect of the obesity epidemic in a nationwide cohort. A description of the ODM1 and ODM2 population has been published previously [29].

### Statistical analysis

The longitudinal analyses fitted smooth, flexible curves with a random-effects model to estimate the growth trajectory of OGDM, and the subgroups non-LGA OGDM and LGA OGDM. The mixed model addressed the correlation of repeated height and BMI SDS measurements obtained within the same child, as well as time-independent variables (maternal age at delivery, parity, educational level, employment hours, marital status, ethnicity, breastfeeding, preconception HbA_1c_, mean pregnancy HbA_1c_, paternal BMI, paternal ethnicity or paternal diabetes) and accommodates to the available values in the dataset. Fixed effects included the covariates maternal diabetes type (GDM), LGA (yes, no), time (age in years) and the interaction between time and maternal diabetes type to show increases or decreases in growth over time. Random effects were intercept and time. Potential confounders were the previously mentioned time-independent variables; these were labelled as covariates in a sensitivity analysis. If the addition of a covariate to the model changed the estimate by more than 10%, we considered this a confounder. In a next step, we checked whether these potential confounders changed the model by visual inspection of the graphs.

Given the known rapid decreases in BMI SDS during the first year of life in LGA infants, in both diabetic and non-diabetic populations [30–33], we separately analysed the growth trajectories in the first year of life and the years thereafter.

In a model with the factors as fixed effects and random effects (mentioned before), the models were examined using the Akaike’s information criterion and Bayesian information criterion. The best model fit had the lowest Akaike’s and Bayesian information criteria, which included a linear and square interaction of diabetes with age, with intercept and age as a random effect, to determine the trajectories for BMI and height SDS. Consequently, for the growth SDS points in the square model, the values of OGDM, non-LGA OGDM and LGA OGDM were modelled as follows:

$$ \mathrm{SDS}=\mathrm{Intercept}+{\beta}_{0\mathrm{ij}}+{\beta}_{1\mathrm{ij}}\ \left(\mathrm{age}\right)+{\beta}_{2\mathrm{ij}}\ {\left(\mathrm{age}\right)}^2 $$

where *β*_0_ represents the intercept, *β*_i_ is the diabetes type (e.g. maternal GDM), *β*_j_ is LGA and age is offspring age in years. Data were analysed using IBM SPSS Statistics version 23.0 for Mac (released 2013; Armonk, NY, USA) and Microsoft Excel for Mac2011 (Impressa Systems, Santa Rosa, CA, USA). Software prepared by the Dutch Growth Research Foundation (Growth Analyser 3.5; Rotterdam, the Netherlands; https://growthanalyser.org/), was used to calculate height SDS using the 2009 data from the Fifth Dutch Growth Study [26] and BMI SDS using the 1980 Dutch nationwide data, which are used as normative standards for present day Dutch children [27].

## Results

### Population characteristics

From 1990 to 2006, 468 offspring and their 406 mothers were identified; efforts were made to contact all parents through mail, telephone and e-mail but 58% of the mothers (*n* = 235) were untraceable as they had moved to an unknown address or their telephone had been disconnected. Another 78 (19%) women refused further participation, resulting in informed consent being provided for 104 children (22%) from 93 mothers (23%). From five women, infants from two consecutive pregnancies were included and there were six dichorionic diamniotic twins. We performed an additional analysis after randomly selecting only one of each twin pair. Since this showed comparable results to the analyses using both twins, we decided to include all twins on which we had data. One infant with trisomy 21 was excluded; there were no infants with major congenital malformations in the study population. Maternal BMI and age were not different between responders and non-responders. However, ethnicity affected the response: more Dutch women of European descent were in the responder group and more women of Mediterranean descent were in the non-responder group.

The baseline characteristics of the 93 mothers and 104 infants included in the study are displayed in Table 1. The mean ± SD number of measurements of height and weight per child was 7.4 ± 2 between birth and 14 years of age, with a total of 771 measurements. Median maternal (interquartile range) BMI was 25.8 (7.8) kg/m^2^, with 82% of the women being of European descent; paternal BMI was 25.5 (3.8) kg/m^2^, with 87% being of European descent. Forty-nine per cent of offspring were female sex. Median birthweight was 3530 (725) g, with 24% being LGA. Treatment with insulin was given to women during 50 (48%) of the 104 pregnancies; for the remainder, only dietary advice was given. Five (4.8%) infants were small for gestational age (birthweight ≤10th percentile).

**Table 1:** Baseline characteristics of infants and parents in the study population

Characteristic	OGDM
Infants	
Infants, *n*	104
Maternal BMI, kg/m^2^	25.8 (7.8)^a^
Maternal age at delivery, years	34 ± 4
Multiparous, *n* (%)	66 (64)
OGTT performed in pregnancy, *n* (%)	82 (79)
Mean pregnancy HbA_1c_, mmol/mol	39 ± 5.5
Mean pregnancy HbA_1c_, %	5.8 ± 0.5^b^
Insulin treatment in pregnancy, *n* (%)	50 (48)
Pre-eclampsia, *n* (%)	7 (7)
Caesarean section, *n* (%)	43 (41)
Gestational age at delivery, weeks	39 (2)
Female sex, *n* (%)	51 (49)
Birthweight, g	3530 (725)
LGA, *n* (%)	25 (24)
Birthweight *z* score	0.57 (1.3)
Neonatal admissions to medium or intensive care, *n* (%)	55 (53)
Breastfeeding at 1 week, *n* (%)	61 (73)^c^
Conditional target height boys, cm	181.7 ± 4.3
Conditional target height girls, cm	169.7 ± 3.9
Parents	
Mothers, *n*	93
Maternal ethnicity European descent, *n* (%)	76 (82)
Paternal ethnicity European descent, *n* (%)	81 (87)
Current paternal BMI, kg/m^2^	25.5 (3.8)^c^
Maternal education (university of applied sciences), *n* (%)	24 (29)^a^
Maternal full-time employment, *n* (%)	14 (17)^a^

Values are mean ± SD or median (interquartile range), unless stated otherwise

^a^10 missing values because of missing questionnaire data

^b^63 missing values because HbA_1c_ was not routinely analysed in pregnancy

^c^20 missing values because of missing questionnaire data

### Growth trajectories: age 1–12 months

**Fig. 1:**
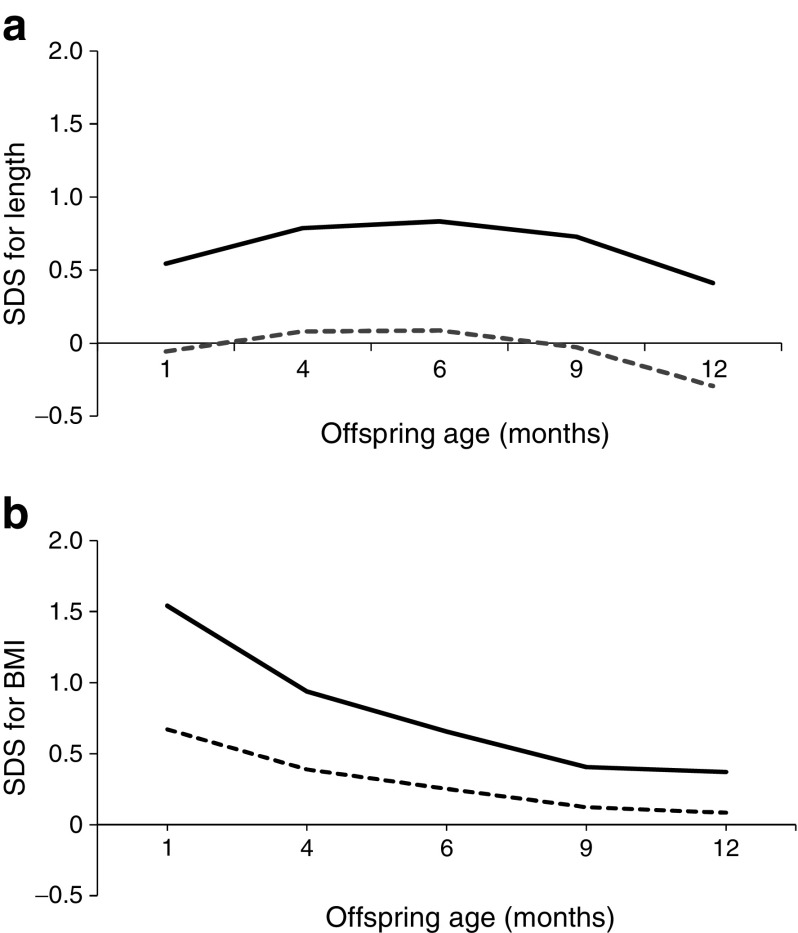
Mixed model for (**a**) length SDS and (**b**) BMI SDS for non-LGA (dashed lines) and LGA (solid lines) OGDM at 1–12 months of age

During the first year of life, the length of the newborns did not differ significantly between non-LGA and LGA infants, although LGA OGDM were slightly longer than non-LGA offspring (Fig. 1a). BMI SDS decreased in both non-LGA and LGA OGDM over the first year of life, with the LGA OGDM having a slightly higher (but not significantly different) BMI SDS than non-LGA infants (Fig. 1b).

### Growth trajectories: age 1–14 years

#### Height

**Fig. 2:**
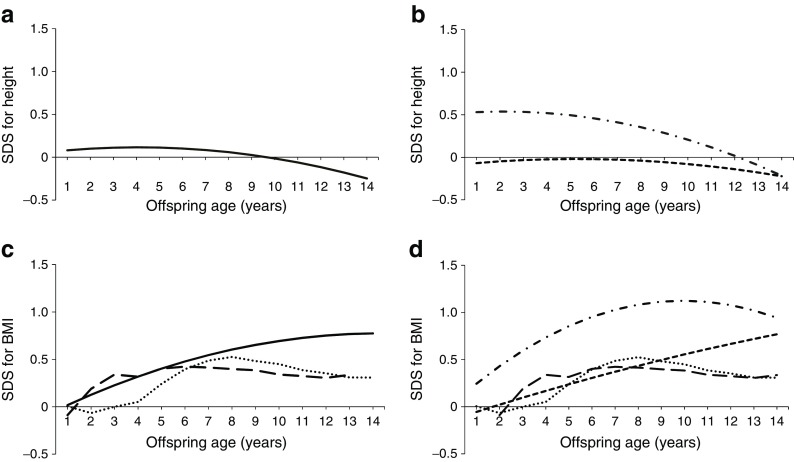
(**a**, **b**) Mixed model for height SDS (**a**) for all OGDM and (**b**) for non-LGA and LGA OGDM at age 1–14 years. (**c**, **d**) Mixed model for BMI SDS (**c**) for all OGDM at age 1–14 years and (**d**) for non-LGA and LGA OGDM at age 1–14 years. Both (**c**) and (**d**) also show reference values from the 2009 reference population for Dutch boys and girls (reference values adapted from Schonbeck et al [28], based on values from Cole and Roede [27]). Solid lines, all OGDM; short dashed lines, non-LGA OGDM; dashed-dotted lines, LGA OGDM; dotted lines, reference values for Dutch boys; long dashed lines, reference values for Dutch girls

The height SDS for OGDM from age 1 to age 14 years was similar to that found in the 2009 Dutch Growth Study (SDS 0), with a slight decrease in early adolescence (Fig. 2a). Although LGA OGDM were slightly taller than non-LGA OGDM, the difference was not statistically significant (Fig. 2b).

#### Comparison with the 2009 (Fifth) Dutch Growth Study

In Fig. 2c, d the BMI SDS is depicted from age 1–14 years, together with that of the 2009 reference population. It was not possible to make statistical comparisons between data from the current study and data from the 2009 nationwide (Fifth) Dutch Growth Study because of differences in definitions and methodology (as outlined in the Discussion). However, visual comparison revealed that, until late childhood (age 8–10 years), the BMI SDS for the total group was similar to that for the 2009 (Fifth) Dutch Growth Study, whereas, after this age, the BMI SDS of the current population was higher (Fig. 2c).

#### BMI

Subgroup analysis showed different growth trajectories between LGA and non-LGA OGDM, but the difference did not reach statistical significance (Fig. 2d, *p* = 0.07). LGA OGDM had a higher BMI SDS at 1 year of age and their BMI SDS continued to increase until late childhood (age 8–10 years), plateauing thereafter; non-LGA OGDM showed a steady increase in BMI SDS up to age 14 years, a BMI trajectory resembling that of Dutch boys and girls of the 2009 population.

The sensitivity analyses included examination of each of the following covariates separately: maternal age at pregnancy; parity; maternal educational level and employment type; maternal and paternal ethnicity; maternal prepregnancy BMI; and current paternal BMI. None of these factors had a statistically significant effect on the slope of the models.

Comparable results were also obtained in analyses performed after excluding twins and in nulliparous women only. Therefore, all of these infants were included in the final analyses. A subgroup analysis for OGDM of European descent (82%) vs non-European descent was not performed due to great ethnic heterogeneity in the latter group.

#### Comparison with ODM1 and ODM2

**Fig. 3:**
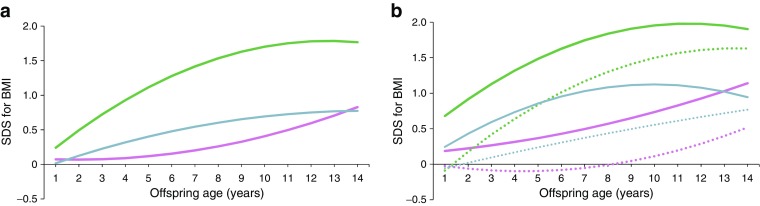
(**a**) Mixed model for BMI SDS for ODM1, ODM2 and OGDM aged 1–14 years. Blue line, OGDM; pink line, ODM1; green line, ODM2. (**b**) BMI SDS for non-LGA and LGA ODM1, ODM2 and OGDM aged 1–14 years. Reference values (ODM1 and ODM2) for BMI SDS for ages 1 to 14 years are adapted from the 2009 nationwide study for Dutch boys and girls [28], based on values from Cole and Roede [27]). Blue line, LGA OGDM; blue dotted line, non-LGA OGDM; pink line, LGA ODM1; pink dotted line, non-LGA ODM1; green line, LGA ODM2; green dotted line, non-LGA ODM2

Figure 3 shows the growth trajectories of OGDM together with those previously reported for ODM1 and ODM2. The latter data were obtained using the same methodology [29]. The LGA and non-LGA OGDM subgroups both had a lower BMI SDS than the equivalent ODM2 subgroups but a higher BMI than the ODM1 subgroups. Between the ages of 1 and 14 years, non-LGA OGDM were comparable with non-LGA ODM1 and the 2009 Dutch Growth Study (shown in Fig. 2), suggesting that the risk for obesity in these subgroups is not increased. LGA OGDM showed slightly higher BMI SDS than LGA ODM1 after the age of 1 year, but values were lower than in either of the ODM2 subgroups. Thus ODM2 (LGA and non-LGA) had the highest BMI SDS.

## Discussion

This study shows that non-LGA and LGA OGDM display different BMI SDS trajectories during childhood, both groups reaching slightly higher values than those of the 2009 (Fifth) Dutch Growth Study. Although LGA OGDM had the higher scores, both subgroups of OGDM had a mean BMI SDS of less than +1 SD when they reached early adolescence, indicating that their risk for becoming overweight in the short term is likely to be limited. The height SDS for non-LGA and LGA OGDM was comparable with that of the 2009 background population. In both the Dutch Growth Study and our OGDM group, BMI SDS compared with the 1980 nationwide study was increased, suggesting a gradual shift in offspring BMI reference values and an increased incidence of overweight/obesity in both the reference group and the OGDM since the 1980s [28].

It is becoming increasingly clear that a hyperglycaemic intrauterine environment is responsible for an increased risk of diseases in childhood and later adulthood. The relatively overnourished offspring of gestational diabetic pregnancies are prone to later development of obesity and diabetes. This is due, not only to genetic susceptibility [34], but also to exposure to the abnormal intrauterine environment with potential for epigenetic changes in the fetal phenotype [35]. These intrauterine phenomena contribute to childhood obesity and possibly to obesity and diabetes in adulthood and an increased risk for cardiovascular disease [36, 37]. Thus, it is evident that preventive strategies are needed. This matter is intuitively most relevant for offspring likely to be most at risk: those born LGA. Therefore, we analysed the growth trajectories of non-LGA and LGA OGDM separately.

Several cross-sectional studies on OGDM have found evidence for overweight/obesity in this population [7, 9–13]. However, although the available longitudinal BMI data in OGDM indicate a higher childhood BMI [17, 19, 20], with sex and maternal prepregnancy BMI as significant contributing factors [18, 21], results cannot be compared due to differences in methodology. In the only other longitudinal study in which LGA and non-LGA infants were studied separately, at age 4–7 years LGA OGDM were found to have a higher BMI than either non-LGA OGDM or LGA offspring from women without diabetes [16]. In a cross-sectional prospective cohort of 6- to 11-year old infants, no difference in BMI was found between LGA and non-LGA OGDM and the offspring of mothers without diabetes in pregnancy [38].

We found that maternal BMI did not influence the BMI SDS trajectory of OGDM. This is in contrast to most studies in which maternal BMI was taken into account [8, 9, 17, 22, 39, 40] and might be due to the relatively low incidence of maternal obesity in our study group (mean BMI, 25.8 kg/m^2^; 30.9% overweight [BMI 25–29.9 kg/m^2^] and 22% obese [BMI ≥30 kg/m^2^] [9, 22, 40]. This is relevant given the higher BMI in most GDM studies from a range of countries [9, 20]. Apparently, this profile of women with GDM is common in our region and may represent a clinically different entity (with a different outcome for their offspring) when compared with other studies. Previous studies have shown that in 16–17-year-old adolescents, the relationship between maternal gestational diabetes and offspring BMI was attenuated after correction for maternal prepregnancy BMI [39] and weight gain in pregnancy [8]. Furthermore, offspring of mothers with gestational diabetes and a normal BMI did not show an increase in obesity, in contrast to offspring of obese mothers with gestational diabetes [9, 40]. Maternal obesity is, therefore, a strong predictor of childhood obesity [14] and a higher childhood BMI is related to maternal BMI [21] in pregnancies complicated by GDM. In our cohort of mothers with GDM, the relatively low maternal BMI may explain the limited effect on childhood BMI.

In the literature we did not find any studies comparing the growth trajectories of OGDM with those of ODM1 and ODM2 separately. Such a comparison is essential when studying (epi)genetic pathways of obesity. Figure 3 clearly shows that ODM2 have the highest risk of childhood obesity. The most striking difference between the GDM, type 1 diabetes and type 2 diabetes groups was the maternal BMI, which was higher in women with type 2 diabetes (31 kg/m^2^), compared with women with type 1 diabetes (24 kg/m^2^) and those with GDM (26 kg/m^2^). This highlights the importance of maternal BMI with respect to infant growth.

Only a few studies are available that examine a heterogenic group consisting of ODM1, ODM2 and OGDM [15, 41–43]; ODM1 and OGDM have been compared as separate groups in only two studies. Overweight rates in OGDM and ODM1 were twice those of the background population [44], with a higher risk for obesity at age 18–20 years in OGDM and only a weak association for ODM1 [45].

### Methodological considerations

This study was performed at a single centre, where pregnant women with GDM were diagnosed and treated according to the same protocol.

A longitudinal control group was not available for comparison. We could, however, compare our data with nationwide data from the Dutch population [26–28]. The reported BMI and height SDS were calculated based on these population values and the latter were plotted as graphs for comparison [28]. Unfortunately, it was not possible to calculate statistical significance between the OGDM subgroups and the nationwide population because the complete set of raw (cross-sectional) data from the Dutch Growth Study was not available for analysis.

In this study, we only received informed consent from 23% of all eligible mothers. The response rate was lower than that reported in the literature (ranging from 46% to 66% [10, 18, 20, 39]; although response rates were not reported in most studies), illustrating that this cohort is hard to trace and recruit. This issue is present explicitly or implicitly in many studies. A prospective cohort study would be the best way to include and follow-up the offspring of pregnant women with GDM.

In the Netherlands, GDM is most prevalent in the subgroups of the population that also have a high incidence of type 2 diabetes, including natives from Turkey, Morocco, Suriname and the Caribbean islands. These groups were under-represented in the current study, possibly because some of these minority populations are under-represented in our region or because the parents and offspring in these groups are sometimes difficult to trace.

Given the ethnic heterogeneity of our study population, a subgroup analysis was not performed. BMI SDS of OGDM in our cohort might have been higher if more Mediterranean-Dutch children had participated [46]. Although the mean growth trajectories, as depicted in the figures, appeared to be different, statistical significance was not always reached for non-LGA OGDM vs LGA OGDM, which may partly be due to the small numbers of Mediterranean-Dutch children involved.

Tanner stage and onset of puberty were not recorded in the current study and, thus, the impact of this natural process on the current group is unknown. By using SDS values from the nationwide study, in which children at different stages of puberty were included, the effect of puberty on height and BMI SDS trajectory is evened out.

In conclusion, this is the first study to analyse longitudinal growth trajectories in OGDM up to the age of 14 years with separate height and BMI SDS for LGA and non-LGA offspring. Growth trajectories were only slightly above those of the Dutch reference population (e.g. Dutch Growth Study), indicating that the risk of OGDM becoming overweight in adolescence seems limited, at least in a population with a low incidence of maternal obesity. Compared with offspring of women with pregestational diabetes, we showed that ODM2 are at highest risk of becoming overweight in early adolescence, followed by LGA ODM1. Non-LGA ODM1 had a growth trajectory comparable with that of the reference population. This study gives a broad view on the intrauterine origins of adult disease in offspring of women with diabetes. Parents and healthcare workers should carefully consider the risk of obesity in offspring of women with diabetes, especially infants born LGA, and should initiate preventive measures where possible. These may include diet and lifestyle coaching for children and parents.

## Data Availability

The datasets generated during and/or analysed during the current study are available from the corresponding author on reasonable request.

## Abbreviations

GDM: Gestational diabetes
LGA: Large for gestational age
ODM1: Offspring of mothers with type 1 diabetes
ODM2: Offspring of mothers with type 2 diabetes
OGDM: Offspring of mothers with gestational diabetes
SDS: Standard deviation score
